# External Load Evaluation in Elite Futsal: Influence of Match Results and Game Location with IMU Technology

**DOI:** 10.3390/jfmk9030140

**Published:** 2024-08-20

**Authors:** Héctor Gadea-Uribarri, Carlos Lago-Fuentes, Ainhoa Bores-Arce, Víctor Emilio Villavicencio Álvarez, Sergio López-García, Santiago Calero-Morales, Elena Mainer-Pardos

**Affiliations:** 1 Faculty of Education, Universidad Pontificia de Salamanca, 37002 Salamanca, Spain; hgadeauribarri@hotmail.com (H.G.-U.); slopezga@upsa.es (S.L.-G.); 2 Faculty of Health Sciences, European University of Atlantic, 39011 Santander, Spain; carlos.lago@uneatlantico.es (C.L.-F.); ainhoa.bores@uneatlantico.es (A.B.-A.); 3 Department of Human and Social Sciences, Universidad de las Fuerzas Armadas-ESPE, Quito 171103, Ecuador; victoremiliovillavicencio@gmail.com; 4 Health Sciences Faculty, Universidad San Jorge, Autov. A23 Km 299, Villanueva de Gállego, 50830 Zaragoza, Spain; epardos@usj.es

**Keywords:** load monitoring, home advantage, high performance, team sports

## Abstract

The purpose of this study was to assess the external load demands in futsal, considering both home and away matches and their outcomes, in order to plan microcycles throughout the season based on the external load of each match. The external load of 10 players from a First Division team in the Spanish Futsal League was recorded throughout 15 official matches in the first half of the league championship. The players’ external load was monitored using OLIVER devices. To analyse the influence of the match outcome and location on the external load, a univariate general linear model (GLM) analysis was conducted with Bonferroni post hoc. There are no differences between the variables neither comparing results nor location factors, except for accelerations of 2 to 3 m/s^2^ (m) per minute and the number of accelerations of 2 to 3 m/s^2^ per minute, reporting higher value winnings at home than away (*p* < 0.05). The location and results are not factors that influence on external load in futsal matches, except the number and distance performed in accelerations and distance covered at a low to medium speed. These findings are important for planning microcycles and providing the appropriate dosage to each player to achieve optimal performance in matches.

## 1. Introduction

Futsal is a team sport where two teams of five players (four outfield players and a goalkeeper) compete on a 40 × 20 m pitch with 3 × 2 m goals [1,2]. In official FIFA competitions, up to 14 players may be summoned [3]. Time is not stopped for substitutions, which are unlimited, and the clock only stops when the ball is out of play [1,4]. Matches consist of two halves of 20 min each, with a total duration of 75 to 90 min [1,3,5]. Each team may request one timeout per half [3]. Unlimited substitutions maintain a high competitive intensity, with intermittent high-intensity actions such as sprints, accelerations, decelerations, and changes in direction, both in defence and attack [1,4,6,7,8].

In recent years, tracking devices and global positioning systems [GPS] have seen significant improvements in this area, allowing for the monitoring of player load during training sessions or matches [5,9,10,11,12]. Additionally, they provide more valid and reliable data that enable the tracking of the specific demands of team sports during matches, through an analysis of various variables, achieving individualised performance profiles [5,13].

Through the systems previously discussed, significant advancements have been made in movement analysis [14]. These systems offer the ability to assess the physical demands of matches, capturing details of player performance during training sessions or matches [15]. For example, it is important to detail the high-intensity activity performed by the player, as this can vary during a match depending on the player’s involvement [16].

Technological advancements in this field make it easier and more accurate to understand and quantify the demands in matches, allowing for better control of external loads. This assists in creating training sessions targeted at these real demands, improving player performance and reducing the risk of injury [6,11,17]. It ensures that objective decisions are made about a player’s availability, considering these two aspects [18]. The match is the most demanding session, hence the importance of load management during the game. With this load control, physical goals for the player can be adjusted throughout the week or the necessary recovery sessions can be planned [8,19].

On the other hand, we must note that in team sports such as football, basketball, or handball, there is already a substantial body of literature on competitive demands [1]. In various studies of sports other than futsal, it is observed that there are several factors that influence game demands, such as the ranking of the opposing team, the location, or the outcome of the match [20,21,22].

In sports such as football, external load varies according to the result and the location of the match. In the event of a draw, players cover shorter distances and perform fewer accelerations and decelerations compared to a loss or victory [20]. Players perform more accelerations in home matches than in away matches [21]. In professional women’s football, the distance covered is greater when the team loses, with no differences between playing at home or away [6]. In amateur football, the location of the match does not influence the external load [22]. In professional football, a player’s performance is affected by the location, the result of the match, and the duration of the microcycle [9]. Additionally, this influences the planning of the microcycle when considering whether the next match is at home or away [23].

Research in various sports disciplines indicates that the ranking of the opponent, the venue, and the outcome of the match can influence the demands of the game. [6]. In this regard, a recent systematic review highlights the importance of two major factors in sports performance: the outcome and “home advantage” in team sports, both tactically and in terms of the typology of efforts. Thus, the review suggests the need for further research on both factors in team sports, especially in indoor sports [24].

In the case of futsal, recently, the performance analysis of players in matches has been of great interest to various researchers [6]. However, the existing literature is scarce regarding the variations in external load influenced by the outcome or location of the game, only studying the impact of these factors in the technical–tactical realm [7].

Given all of the above, the aim of the present study was to analyse the effects of external load on various variables in futsal matches, taking into account the location and the result, over half a season. In doing so, it is intended to interpret these results to be able to plan microcycles throughout the season according to the external load of each match.

## 2. Materials and Methods

### 2.1. Study Design

This study has an observational longitudinal design spanning the first half of the 2022–2023 season (from September to December 2022). The players were monitored during the first half of the 2022–2023 season of the 1st Division of the National Futsal League, i.e., over 15 official matches resulting in 5 wins, 3 draws, and 7 losses, finishing the first half of the regular league in 9th position (one position away from qualifying for the Spanish Cup).

The external load of the players was monitored using the IMU (Inertial Measurement Unit) OLIVER (Barcelona, Spain) devices. All players wore the IMU OLIVER device on their calf, and the devices were turned on just after the warm-up ended, i.e., between 8 and 10 min before the start of the match. The IMU OLIVER is small-sized (4 × 5 × 1.5 cm) and lightweight (15 g). The actions were recorded by the IMU hardware work at 27 Hz (Recorder and Analyzer, Barcelona, Spain). At the start of the matches, all players placed the IMU OLIVER device on the middle of their dominant leg at the height of the gastrocnemius muscle at the back. At the end of the matches, the players removed the devices, turned them off, and their data were transferred to the respective software for data extraction (TryOliver Platform 2.42) [25]. To avoid possible errors between devices, each player used the same device throughout the first half of the season.

All players were verbally informed about the purpose and procedures of the study, and all signed an informed consent form in accordance with the Declaration of Helsinki, which was approved by the University’s Research Ethics Committee (CEI-35/2022).

### 2.2. Participants

The external load of 10 elite (TIER 3) [26] players from a First Division team in the Spanish Futsal League, two of whom were part of their national teams (age 27.5 ± 7 years, height 1.73 ± 0.05 m, weight 70.1 ± 3.8 kg), was recorded. Goalkeepers were excluded from the study. The average duration of player participation with the clock stopped was 18.15 ± 7.04 min (mean ± standard deviation).

### 2.3. Procedure

Building on previous studies conducted in futsal [1,3,7] that analysed the conditional demands of competition, the variables analysed in this study were total distance (m), walking 0–6 km/h (m), jogging 6.1–12 km/h (m), high-intensity distance 12.1–18 km/h (m), maximum intensity distance >18.1 km/h (m), high acc (m) at high intensity (>2 to 3 m/s^2^) and maximum intensity (>3 to 10 m/s^2^), high dec (m) at high intensity (>−2 to −3 m/s^2^) and maximum intensity (>−3 to −10 m/s^2^), number of accelerations at high intensity (>2 to 3 m/s^2^) and maximum intensity (>3 to 10 m/s^2^), number of decelerations at high intensity (>−2 to −3 m/s^2^) and maximum intensity (>−3 to −10 m/s^2^), and number of sprints at high intensity (>12 km/h) and maximum intensity (>18 km/h), and MAX Speed (km/h). Once the matches were played, the data were categorised based on two variables—home/away and result: win/draw/loss—considering some previously published scientific studies [6,27,28].

### 2.4. Statistical Analysis

Firstly, the normality of the data was checked using the Shapiro–Wilk test and the results revealed that all the variables presented a normal distribution of data. Also, to properly compare the data, absolute and relative (effective playing time—clock time) values of each variable were calculated [13,29], except max speed. Subsequently, a descriptive statistic of the variables was carried out, and all data were presented as mean (M), standard deviation (SD), and confidence interval (IC 90%). For the analysis of the influence of the result and the location of the match on the external load, a univariate general linear model (GLM) analysis was conducted. The external load variables were taken as dependent variables, the location of the match was taken as fixed factor and the result of the match as a random factor, and the Bonferroni post hoc test was applied. The level of statistical significance was set at *p* < 0.01. All statistical analyses were performed using the SPSS statistical package (v.29.0; IBM Corporation, Armonk, NY, USA).

## 3. Results

Table 1 displays the descriptive statistics for the total and per-minute normalised data for each of the external load variables, which show high values of total and relative distance in the matches (3728.2 ± 1152.8 m and 221.6 ± 53.1, respectively).

Table 2 shows the external demands of the match considering the complete game, as well as home or away and the result separately (i.e., average values of all games). No significant differences were observed between the different conditions (*p* > 0.05), except the distance covered from 0 to 6 km/h, performing higher distances losing away compared than losing at home (*p* < 0.05).

Table 3 presents the external load variables normalised according to the result and location of the match per minute. The results of the study revealed that the total distance covered per minute was significantly higher when winning at home than away (*p* = 0.041), as well as distance at low to medium distances (*p* < 0.05). Also, the number and distance of decelerations at medium intensity showed higher values when winning at home than away (*p* < 0.05). The rest of variables did not show significant differences, neither by match result nor game location (*p* > 0.05).

## 4. Discussion

The aim of the present study was to analyse the effects of external loading on various variables in futsal matches, considering the venue and the result, over half a season. The main findings are as follows: with respect to total data, most variables have the highest data at home when drawing or winning and away when winning. With respect to data per minute, most variables have the highest data at home when winning. In addition, the total distance and distance covered per minute at low and medium speeds are significantly higher when winning at home than winning away. Nonetheless, the rest of the variables were not influenced regarding match result nor game location.

Regarding the home/away effect, several studies have addressed this topic. A study in the NBA states that teams score an average of 0.13 points more per minute in home games [30]. Another study on handball examines this issue and concludes that the home advantage in handball is approximately 61.3% [31]. A study on football in the Brazilian league indicates a home advantage, with home teams scoring more goals than away teams in the last 11 seasons. In our study, there are more wins when playing at home than when playing away, which is consistent with the findings of other sports and leagues. This suggests that the psychological and environmental factors contributing to the home advantage, such as crowd support, familiarity with the venue, and reduced travel fatigue, are likely universal across different sports [32].

Regarding the average total distance, recent studies have recorded distances of 3060 m, 3868 m, and 3749 m in both the Spanish and Portuguese leagues [1,6,13]. In this study, the average total distance is 3728 m ± 1152 m. Compared to another study in football [22], the total distance (m) in a football match is 10,265 m. In comparison with another indoor sport such as basketball, a systematic review [33] indicates that the distance covered in a match ranges from 6279 m to 7558 m. This suggests that the findings of the present study are in line with the scientific literature in futsal, being one of the sports in which the least total distance is covered.

On the other hand, the average high-intensity distance is another critical performance factor in team sports. In futsal, studies indicate that 675.3 m are covered in a match [13], and in this article, it was 691.18 m. Additionally, for the average maximum speed distance, several articles range from 239.33 m [1] to 134.9 m [13], while in this article, it was 223.51 m. This may be due to competitive differences between the different leagues, such as the Portuguese and the Spanish leagues, with the Spanish league showing a higher level of conditional demands compared to the Portuguese league.

Focusing on the number of accelerations > 2 m/s^2^ (n) and the number of decelerations > −2 m/s^2^ (n), there are studies in futsal that report 130.33 accelerations and 125 decelerations [1]. In this study, there were 135.06 accelerations and 136.56 decelerations. In football, the number of accelerations > 2 m/s^2^ (n) and the number of decelerations > −2 m/s^2^ (n) are 250 accelerations and 223 decelerations [22]. Considering the competition time per player, it is observed that futsal requires a very high volume of accelerations and decelerations for the competition time, with between 7 and 8 accelerations and decelerations per minute.

Taking into account the normalised per minute data of the different variables, the number of accelerations per minute > 2 m/s^2^ (n/min) and the number of decelerations per minute > 2 m/s^2^ (n/min), we found one article on indoor football providing relevant information of 3.93 accelerations per minute and 3.8 decelerations per minute [1], and another article that reports 5 accelerations and decelerations per minute [13]. In the present study, there were 7.87 accelerations per minute and 8 decelerations per minute.

In relation to the factors analysed in this study, it is observed that, in general, neither the result nor the location of the competition influences the key performance indicators in futsal, except for total distance per minute (m), distance at [0–6] km/h (m) per minute, distance at [6.1–12] km/h (m) per minute, number of accelerations at high intensity [2–3] m/s^2^ per minute, number of decelerations (>−2 m/s^2^) per minute, decelerations at high intensity [−3–−2] m/s^2^ (m) per minute, and number of decelerations at high intensity [−3–−2] m/s^2^ per minute, which are higher in matches won at home compared to matches won away. This may be due to tactical decisions by the coaching staff, proposing a more conservative pattern of play when playing away compared to home matches, as has been observed in other sports such as football [34].

On the other hand, considering the location of the match, we find futsal studies indicating that the average total distance covered by the team when playing at home was 3757 m, and when playing away was 4036 m [6]. In this study, the average total distance covered by the team when playing at home was 3731.16 m, and when playing away was 3707.18 m.

If we consider the match result, we find futsal studies where the average total distance covered by the team when winning was 3846 m, and when losing was 3990 m [6]. In this study, the average total distance covered by the team when winning was 3849.86 m, and when losing was 3631.02 m. However, in the present research, there were no significant differences in relation to the total distance, which may be due to the total number of matches analysed [1,6,13,29], which should be larger in future research, as well as the need to analyse the game model given its clear influence on performance parameters.

Despite the findings of this research, it is not without limitations. Thus, this study is limited by only covering the first half of the league, and it would have been interesting to compare the external load against each team in the home and away matches. Also, this study only recorded data from one team, attending to the complexity of achieving these data from different teams [1,6,13,29]; however, the sample size analysed is in line with previous research on competitive demands in team sports. Furthermore, the scarcity of scientific literature in futsal on competitive demands and the use of the same variables limits the discussion and comparison of the findings. Nevertheless, this opens a line of research to continue investigating the influence of both factors studied in futsal, both in the Spanish league and in other competitions of a similar competitive level. Finally, the absence of contextual records related to the audience limits the interpretation of the influence of the home advantage factor [34], so future research should include factors such as attendance, audience, refereeing actions, etc., to model a comprehensive analysis of this decisive factor.

These findings are of great relevance to physical trainers and coaching staff as they provide a diagnosis of the competitive demands faced by players in high-pressure environments. This allows for the adjustment of training loads in the microcycle leading up to the competition to ensure optimal performance, as well as guiding effective post-competition recovery strategies, especially considering the high neuromuscular demands in matches, both at home and away. Thus, the future line of research will be to design microcycles throughout the season, taking into account the external load of each match and also considering player positions to enhance the precision of training and recovery protocols.

## 5. Conclusions

In relation to the objective of this study, the relative variables of total distance per minute (m), distance at [0–6] km/h (m) per minute, distance at [6.1–12] km/h (m) per minute, number of high-intensity accelerations [2,3] m/s^2^ per minute, number of decelerations (>−2 m/s^2^) per minute, high-intensity decelerations [−3–−2] m/s^2^ (m) per minute, and number of high-intensity decelerations [−3–−2] m/s^2^ per minute are significantly higher when winning at home compared to winning away. However, most absolute variables were not influenced by the match result or the venue, except for the distance at [0–6] km/h (m). Therefore, futsal can be considered a sport in which normalised data per minute should be given more importance than absolute data.

## Figures and Tables

**Table 1:** Descriptive statistics for total and per-minute normalised data for each variable.

	Absolute	Relative (per Minute)
	M ± SD	M ± SD
Total distance (m)	3728.2 ± 1152.8	221.6 ± 53.1
[0–6] km/h (m)	1191.8 ± 308.2	74.6 ± 30.4
[6.1–12] km/h (m)	1363.4 ± 441.6	79.4 ± 13.7
[12.1–18] km/h (m)	691.1 ± 2617	40.8 ± 12.3
[18.1–3600] km/h (m)	223.5 ± 125.9	12.5 ± 5.91
High Acc quantity (>2 m/s^2^)	135.0 ± 45.4	7.8 ± 1.6
High Acc (m) (>2 m/s^2^)	534.5 ± 184.8	31.1 ± 6.88
[2–3] m/s^2^ (m)	332.8 ± 111.0	19.5 ± 4.48
[3–10] m/s^2^ (m)	201.7 ± 85.4	11.6 ± 3.59
[2–3] m/s^2^ quantity	90.8 ± 30.0	5.32 ± 1.17
[3–10] m/s^2^ quantity	44.2 ± 18.3	2.55 ± 0.75
High Dec quantity (>−2 m/s^2^)	136.5 ± 49.4	8 ± 2.13
High Dec (m) (>−2 m/s^2^)	510.4 ± 178	29.9 ± 7.41
[−3–−2] m/s^2^ (m)	315.8 ± 115.0	18.7 ± 5.81
[−10–−3] m/s^2^ (m)	194.6 ± 77.4	11.1 ± 3.08
[−3–−2] m/s^2^ quantity	90.4 ± 35.64	5.36 ± 1.85
[−10–−3] m/s^2^ quantity	46.1 ± 18.1	2.66 ± 0.71
Maximum intensity sprints (>18 km/h) (n)	33.3 ± 17.3	1.88 ± 0.78
High-intensity sprints (>12 km/h) (n)	93.2 ± 35.5	5.44 ± 1.43
Maximum Speed(km/h)	24.78 ± 1.8	-

M = mean; SD = standard deviation.

**Table 2:** Differences in absolute external load variables according to match location and outcome.

	Home						Away						
	Loss		Draw		Win		Loss		Draw		Win		*p*
	M ± SD	IC (90%)	M ± SD	IC (90%)	M ± SD	IC (90%)	M ± SD	IC (90%)	M ± SD	IC (90%)	M ± SD	IC (90%)	
Total distance (m)	3481.48 ± 223.63	3110.9–3852.06	3950 ± 290.5	3468.6–4431.4	3762.96 ± 223.63	3392.38–4133.54	3780.56 ± 193.67	3459.62–4101.49	3422.22 ± 387.33	2780.36–4064.09	3918.75 ± 290.5	3437.35–4400.15	0.343
[0–6] km/h (m)	1086.42 ± 58.5	989.47–1183.37	1228.99 ± 76	1103.05–1354.93	1228.66 ± 58.5	1131.71–1325.61	1265.37 ± 50.67	1181.41–1349.33	1026.04 ± 101.33	858.13–1193.96	1198.45 ± 76	1072.51–1324.39	0.027 *
[6.1–12] km/h (m)	1305.89 ± 85.86	1163.61–1448.18	1456.6 ± 111.54	1271.77–1641.43	1388.18 ± 85.86	1245.89–1530.46	1338.2 ± 74.36	1214.98–1461.42	1253.82 ± 148.72	1007.38–1500.27	1443.75 ± 111.54	1258.92–1628.58	0.492
[12.1–18] km/h (m)	640.37 ± 50.82	556.15–724.6	739.27 ± 66.02	629.86–848.68	661.77 ± 50.82	577.55–746	703.52 ± 44.01	630.58–776.46	694.64 ± 88.03	548.77–840.52	748.73 ± 66.02	639.32–858.14	0.616
[18.1–3600] km/h (m)	206.14 ± 24.5	165.54–246.75	248.76 ± 31.83	196.02–301.51	213.67 ± 24.5	173.07–254.28	222.61 ± 21.22	187.45–257.77	212.71 ± 42.44	142.38–283.04	252.27 ± 31.83	199.52–305.01	0.531
High Acc quantity (>2 m/s^2^)	130.41 ± 8.81	115.82–145	142.5 ± 11.44	123.54–161.46	142.07 ± 8.81	127.48–156.67	128.31 ± 7.63	115.67–140.94	126.67 ± 15.25	101.39–151.94	143.56 ± 11.44	124.61–162.52	0.759
High Acc (m) (>2 m/s^2^)	511.67 ± 35.86	452.24–571.1	568.94 ± 46.59	491.74–646.14	557.33 ± 35.86	497.91–616.76	513.01 ± 31.06	461.54–564.47	494.99 ± 62.11	392.06–597.92	571.08 ± 46.59	493.88–648.28	0.640
[2–3] m/s^2^ (m)	317.64 ± 21.41	282.15–353.12	350.56 ± 27.82	304.46–396.65	358.36 ± 21.41	322.87–393.84	315.79 ± 18.54	285.06–346.52	301.41 ± 37.09	239.95–362.87	353.48 ± 27.82	307.39–399.58	0.667
[3–10] m/s^2^ (m)	193.99 ± 16.68	166.36–221.62	218.33 ± 21.66	182.43–254.23	199.27 ± 16.68	171.63–226.9	197.16 ± 14.44	173.23–221.1	193.54 ± 28.88	145.68–241.41	217.54 ± 21.66	181.65–253.44	0.637
[2–3] m/s^2^ quantity	87.59 ± 5.79	78–97.19	94.69 ± 7.52	82.22–107.15	98.52 ± 5.79	88.92–108.11	85.25 ± 5.01	76.94–93.56	84.11 ± 10.03	67.49–100.73	95.81 ± 7.52	83.35–108.28	0.844
[3–10] m/s^2^ quantity	42.81 ± 3.57	36.89–48.74	47.81 ± 4.64	40.12–55.51	43.56 ± 3.57	37.63–49.48	43.06 ± 3.1	37.93–48.18	42.56 ± 6.19	32.3–52.81	47.75 ± 4.64	40.06–55.44	0.622
High Dec quantity (>−2 m/s^2^)	134.48 ± 9.68	118.45–150.51	139.69 ± 12.57	118.86–160.52	142.04 ± 9.68	126–158.07	133.33 ± 8.38	119.45–147.22	127.44 ± 16.76	99.67–155.21	140.06 ± 12.57	119.23–160.89	0.897
High Dec (m) (>−2 m/s^2^)	500.7 ± 34.81	443.02–558.37	522.45 ± 45.21	447.52–597.38	526.24 ± 34.81	468.57–583.92	501.86 ± 30.14	451.91–551.81	474.93 ± 60.29	375.03–574.84	527.56 ± 45.21	452.64–602.49	0.843
[−3–−2] m/s^2^ (m)	307.28 ± 22.53	269.94–344.62	323.61 ± 29.27	275.1–372.11	324.4 ± 22.53	287.06–361.74	315.28 ± 19.51	282.94–347.61	298.34 ± 39.03	233.67–363.02	318.94 ± 29.27	270.43–367.44	0.840
[−10–−3] m/s^2^ (m)	193.37 ± 15.09	168.36–218.38	198.82 ± 19.61	166.33–231.31	201.81 ± 15.09	176.8–226.83	186.55 ± 13.07	164.89–208.21	176.53 ± 26.14	133.21–219.85	208.53 ± 19.61	176.04–241.02	0.775
[−3–−2] m/s^2^ quantity	88.59 ± 6.98	77.03–100.16	92.25 ± 9.07	77.23–107.27	94.11 ± 6.98	82.55–105.68	89.64 ± 6.04	79.62–99.65	85.33 ± 12.09	65.3–105.36	90.63 ± 9.07	75.6–105.65	0.891
[−10–−3] m/s^2^ quantity	45.89 ± 3.52	40.05–51.73	48.06 ± 4.58	40.48–55.65	47.93 ± 3.52	42.09–53.76	43.69 ± 3.05	38.64–48.75	42.11 ± 6.1	32–52.22	49.44 ± 4.58	41.85–57.02	0.730
Maximum intensity sprints (>18 km/h) (n)	30.78 ± 3.38	25.18–36.37	36.81 ± 4.39	29.54–44.08	31.7 ± 3.38	26.11–37.3	34.06 ± 2.92	29.21–38.9	30.89 ± 5.85	21.2–40.58	36.69 ± 4.39	29.42–43.96	0.464
High-intensity sprints (>12 km/h) (n)	86.52 ± 6.9	75.08–97.95	100.81 ± 8.96	85.96–115.67	90.26 ± 6.9	78.82–101.69	93.31 ± 5.98	83.4–103.21	93.22 ± 11.95	73.42–113.03	101.88 ± 8.96	87.02–116.73	0.584
Maximum Speed (km/h)	24.82 ± 0.35	24.24–25.39	25.07 ± 0.45	24.32–25.82	25.04 ± 0.35	24.47–25.62	24.25 ± 0.3	23.75–24.75	24.61 ± 0.6	23.62–25.61	25.23 ± 0.45	24.48–25.97	0.580

M = mean; SD = standard deviation; CI = confidence interval. * *p* = 0.022 away loss compared to home loss.

**Table 3:** Differences in relative external load variables according to match location and outcome.

	Home						Away						
	Loss		Draw		Win		Loss		Draw		Win		*p*
	M ± SD	IC (90%)	M ± SD	IC (90%)	M ± SD	IC (90%)	M ± SD	IC (90%)	M ± SD	IC (90%)	M ± SD	IC (90%)	
Total distance (m)	212.81 ± 10.16	195.97–229.64	211.4 ± 13.2	189.53–233.27	240.68 ± 10.16	223.85–257.52	226.74 ± 8.8	212.16–241.32	216.43 ± 17.6	187.27–245.59	206.33 ± 13.2	184.45–228.2	0.076 *^1^
[0–6] km/h (m)	72.38 ± 5.83	62.72–82.04	68.77 ± 7.57	56.22–81.32	84.06 ± 5.83	74.4–93.72	78.15 ± 5.05	69.79–86.52	69.47 ± 10.1	52.74–86.2	63.44 ± 7.57	50.89–75.99	0.097 *^2^
[6.1–12] km/h (m)	77.03 ± 2.6	72.72–81.33	76.97 ± 3.37	71.38–82.56	86.25 ± 2.6	81.94–90.55	79.92 ± 2.25	76.2–83.65	75.48 ± 4.5	68.03–82.94	75.93 ± 3.37	70.34–81.52	0.057 *^3^
[12.1–18] km/h (m)	38.14 ± 2.39	34.17–42.1	39.44 ± 3.11	34.29–44.6	41.48 ± 2.39	37.52–45.45	42.63 ± 2.07	39.2–46.07	44.53 ± 4.15	37.66–51.41	39.66 ± 3.11	34.5–44.81	0.397
[18.1–3600] km/h (m)	12.08 ± 1.16	10.16–14	12.38 ± 1.5	9.88–14.87	12.63 ± 1.16	10.72–14.55	12.57 ± 1	10.9–14.23	13.49 ± 2.01	10.17–16.82	13.1 ± 1.5	10.61–15.59	0.974
High Acc quantity (>2 m/s^2^)	7.73 ± 0.3	7.23–8.23	7.51 ± 0.39	6.86–8.17	8.69 ± 0.3	8.18–9.19	7.69 ± 0.26	7.25–8.12	7.64 ± 0.53	6.76–8.51	7.63 ± 0.39	6.98–8.29	0.217
High Acc (m) (>2 m/s^2^)	30.42 ± 1.32	28.24–32.6	29.93 ± 1.71	27.1–32.76	34.11 ± 1.32	31.93–36.29	30.67 ± 1.14	28.78–32.56	30.14 ± 2.28	26.36–33.91	30.39 ± 1.71	27.56–33.22	0.322
[2–3] m/s^2^ (m)	19.04 ± 0.84	17.65–20.43	18.64 ± 1.09	16.83–20.44	22.15 ± 0.84	20.76–23.54	18.96 ± 0.73	17.76–20.17	18.43 ± 1.45	16.02–20.83	18.72 ± 1.09	16.92–20.52	0.142
[3–10] m/s^2^ (m)	11.37 ± 0.7	10.21–12.54	11.29 ± 0.91	9.78–12.81	11.98 ± 0.7	10.81–13.15	11.71 ± 0.61	10.7–12.72	11.71 ± 1.22	9.69–13.72	11.67 ± 0.91	10.15–13.18	0.892
[2–3] m/s^2^ quantity	5.22 ± 0.22	4.86–5.58	5.03 ± 0.28	4.57–5.5	6.07 ± 0.22	5.71–6.43	5.13 ± 0.19	4.82–5.44	5.08 ± 0.38	4.46–5.7	5.07 ± 0.28	4.6–5.54	0.095 *^6^
[3–10] m/s^2^ quantity	2.5 ± 0.15	2.26–2.75	2.48 ± 0.19	2.16–2.8	2.62 ± 0.15	2.37–2.86	2.55 ± 0.13	2.34–2.77	2.56 ± 0.26	2.13–2.98	2.56 ± 0.19	2.24–2.88	0.924
High Dec quantity (>−2 m/s^2^)	7.8 ± 0.41	7.13–8.47	7.39 ± 0.53	6.52–8.27	8.9 ± 0.41	8.23–9.57	8.13 ± 0.35	7.55–8.71	7.48 ± 0.7	6.32–8.65	7.43 ± 0.53	6.55–8.3	0.100 *^4^
High Dec (m) (>−2 m/s^2^)	29.1 ± 1.41	26.76–31.44	27.78 ± 1.83	24.74–30.82	32.76 ± 1.41	30.42–35.1	30.6 ± 1.22	28.58–32.63	28.18 ± 2.44	24.12–32.23	28.04 ± 1.83	25.01–31.08	0.108
[−3–−2] m/s^2^ (m)	18.05 ± 1.11	16.21–19.89	17.15 ± 1.44	14.76–19.54	20.71 ± 1.11	18.87–22.55	19.45 ± 0.96	17.86–21.04	18.01 ± 1.92	14.83–21.2	16.83 ± 1.44	14.44–19.22	0.070 *^5^
[−10–−3] m/s^2^ (m)	11.04 ± 0.6	10.06–12.03	10.63 ± 0.77	9.34–11.91	12.05 ± 0.6	11.06–13.04	11.15 ± 0.52	10.3–12	10.16 ± 1.03	8.45–11.87	11.21 ± 0.77	9.93–12.49	0.744
[−3–−2] m/s^2^ quantity	5.18 ± 0.35	4.6–5.77	4.87 ± 0.46	4.11–5.64	6.04 ± 0.35	5.45–6.62	5.52 ± 0.31	5.01–6.03	5.06 ± 0.61	4.04–6.07	4.78 ± 0.46	4.01–5.54	0.089 *^7^
[−10–−3] m/s^2^ quantity	2.62 ± 0.14	2.39–2.85	2.61 ± 0.18	2.31–2.91	2.86 ± 0.14	2.63–3.09	2.61 ± 0.12	2.41–2.81	2.43 ± 0.24	2.03–2.82	2.65 ± 0.18	2.35–2.95	0.753
Maximum intensity sprints (>18 km/h) (n)	1.79 ± 0.15	1.54–2.04	1.88 ± 0.2	1.55–2.21	1.89 ± 0.15	1.64–2.14	1.93 ± 0.13	1.71–2.14	1.89 ± 0.26	1.45–2.32	1.91 ± 0.2	1.58–2.24	0.915
High-intensity sprints (>12 km/h) (n)	5.07 ± 0.28	4.61–5.53	5.31 ± 0.36	4.71–5.9	5.58 ± 0.28	5.12–6.04	5.61 ± 0.24	5.21–6.01	5.86 ± 0.48	5.07–6.66	5.35 ± 0.36	4.75–5.94	0.367
Maximum Speed (km/h)	24.82 ± 0.35	24.24–25.39	25.07 ± 0.45	24.32–25.82	25.04 ± 0.35	24.47–25.62	24.25 ± 0.3	23.75–24.75	24.61 ± 0.6	23.62–25.61	25.22 ± 0.45	24.48–25.97	0.547

M = mean; SD = standard deviation; CI = confidence interval; *^1^ *p* = 0.041 home win compared to away win; *^2^ *p* = 0.033 home win compared to away win; *^3^ *p* = 0.017 home win compared to away win; *^4^ *p* = 0.028 home win compared to away win; *^5^ *p* = 0.035 home win compared to away win; *^6^ *p* = 0.006 home win compared to away win; *^7^ *p* = 0.032 home win compared to away win.

## Data Availability

The data from this research can be made available by the corresponding author following a justified request. Due to privacy concerns, the data are not accessible to the public.
